# A Rare Case of Empyema Complicated With Bronchopleural Fistula Secondary to Mucormycosis in a Young Immunocompromised Diabetic Patient With COVID-19

**DOI:** 10.7759/cureus.26635

**Published:** 2022-07-07

**Authors:** Ruby Risal, Tahmina Jahir, Ratul Islam, Pharlin Noel, Kamal R Subedi, Ahmad Khan, Aneeta Kumari, Marie Schmidt

**Affiliations:** 1 Pulmonary Medicine, Interfaith Medical Center, Brooklyn, USA; 2 Medicine, American University of Antigua, New York, USA; 3 Surgery, Mount Sinai South Nassau Hospital, Oceanside, USA; 4 Department of Medicine, Interfaith Medical Center, Brooklyn, USA; 5 Pulmonary and Critical Care Medicine, Interfaith Medical Center, Brooklyn, USA

**Keywords:** empyema necessitans, diabetic ketoacidosis (dka), “diabetes mellitus”, “mucormycosis”, covid 19

## Abstract

Mucormycosis is an opportunistic fungal infection caused by the zygomycetes Mucor and Rhizopus. Most documented conditions and risk factors that predispose to mucormycosis are uncontrolled diabetes mellitus (DM), with or without ketoacidosis, hematological malignancies (HM), transplantation, immunosuppression, and chronic sinusitis. Pulmonary empyema secondary to Mucor in coronavirus disease 2019 (COVID-19)-infected patients is rarely documented. Here we present an extremely rare case of pulmonary empyema secondary to Mucor infection complicated by bronchocutaneous fistula in a human immunodeficiency virus (HIV)-infected patient in the setting of acute COVID-19 infection.

## Introduction

Mucormycosis, also known as the black fungus, is a life-threatening fungal infection that affects the brain, lungs, eyes, and even sinuses in diabetic or severely immunocompromised individuals [1]. Mucormycosis is a thermotolerant eukaryotic fungus that belongs to the order of Mucorales [2]. Although present for decades, cases of mucormycosis are found in immunocompromised individuals and are caused by the inhalation of filamentous fungi, from the natural environment or in nosocomial settings [3]. It is generally found in patients that contain debilitating conditions such as coronavirus disease 2019 (COVID-19), influenza, uncontrolled diabetes, recent organ transplantation, and also poor hygiene conditions [3]. In a recent report of 275 cases of COVID-associated mucormycosis (CAM), 233 were reported from India and indicated diabetes mellitus as the most frequent risk factor for India [3]. During the second wave of the COVID-19 pandemic, there had been an increased reporting of greater than 9000 cases of invasive mucormycosis post COVID-19 [4]. According to a meta-analysis of 52,916 COVID-19 patients, the incidence of CAM was 50 times higher than the highest known background of mucormycosis cases which is reported at 0.14 cases/1000 patients [3]. In another study, diabetes mellitus had been the underlying disease associated in 54 to 76% of mucormycosis cases and 8 to 22% associated with diabetic ketoacidosis [4]. In a study of 102 mucormycosis cases observed in different regions of India, diabetes mellitus was the most prevalent risk factor in 88.2% of the cases associated with COVID-19 [5]. Mucormycosis, well known for its high mortality rate and poor prognosis, should be treated without delay. We present a rare case of a young poorly controlled diabetic patient with a newly diagnosed acute COVID-19 infection who presented with empyema secondary to mucormycosis which was complicated by bronchopleural fistula. We also reviewed the literature and explored the etiology, risk factors, prognosis, prevention, and treatment options.

## Case presentation

**Figure 1:**
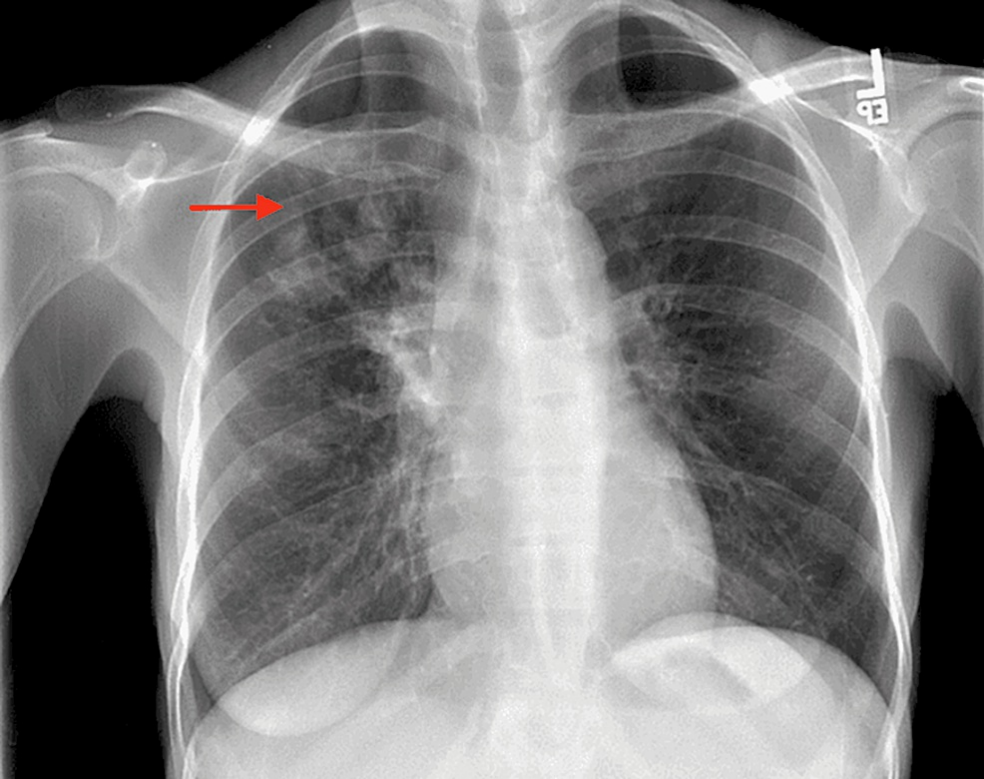
Right upper lobe (red arrow) and perihilar consolidations.

A 34-year-old female with a past medical history of HIV on highly active antiretroviral therapy (HAART), hypertension, newly diagnosed type II diabetes mellitus, bronchial asthma, chronic pancreatitis, dyslipidemia, unvaccinated for COVID-19, presented to the emergency department (ED) with worsening chest pain and dry cough for three days. Chest pain was located on right side, sharp in nature, aggravated by cough and touch but no relieving factor. She denied any fever or shortness of breath. At presentation, the temperature was 98.1F, heart rate of 96 beats per minute, respiratory rate of 18 breaths per minute, blood pressure of 124/86 mm of Hg and saturation of 98% on room air (RA). Review of systems was unremarkable except for cough and chest pain. On physical examination, the chest was clear on auscultation bilaterally. Labs at the time of admission showed leukocytosis of 10.7 × 109/L (reference range: 4.5-11 × 109/L) with a left shift of neutrophils at 77.1% and relative lymphopenia at 9.4%. The chemistry panel shows the following findings: glucose of 455 mg/dl, Na+/K+ of 127/5.5 mmol/L, Cl- of 98 mmol/L, HCO3 of 11 mEq/L, pH 7.28 and anion gap of 18. Blood urea nitrogen (BUN)/creatinine ratio was 9.9/1.04 mg/dL and calcium was 8.9 mg/dL. Liver function tests showed total bilirubin of 0.5 mg/dL, aspartate transaminase of 44 U/L, alanine transaminase of 30 U/L, alkaline phosphatase of 175 U/L, total protein of 8.6 g/dL, albumin of 3.1 m/dL. Polymerase chain reaction (PCR) test for (COVID-19) was negative. Coagulation profile was not done. Chest X-Ray (CXR) on admission revealed right upper lobe and perihilar consolidations (Figure 1). The patient was admitted for diabetic ketoacidosis (DKA) and community-acquired pneumonia (CAP). She was managed by intravenous fluids, insulin drip as per the DKA protocol as well as ceftriaxone and azithromycin for CAP. One set of blood cultures was positive for methicillin-sensitive Staphylococcus aureus (MSSA). However, she left against medical advice before completing the course of treatment and further workup.

**Figure 2:**
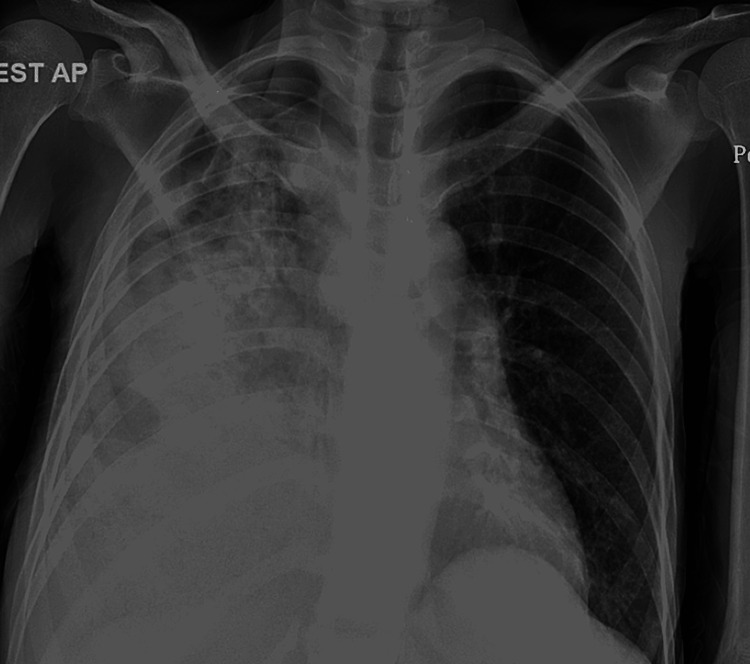
Marked worsening of airspace opacities in the right lung with consolidation in the right mid and lower lung and possible right pleural effusion.

After 10 days she returned to the ED with complaints of nausea, vomiting, and abdominal pain for three days. On arrival, the patient reported feeling depressed and not being compliant with medications. Lab investigations revealed a complete blood count (CBC) with significantly elevated white blood cells (WBCs) of 21.5. General chemistry revealed uncontrolled blood glucose of 380 mg/dL, reduced bicarbonate of 16 mEq/L, and an anion gap of 19. Arterial blood gas (ABG) revealed a pH of 7.38. The patient also tested positive for COVID-19. CD4/helper T cell count of 499 and HIV viral load of under 40. CXR showed worsening of airspace opacities in the right lung consolidation in right mid and lower lung with possible right lung effusion (Figure 2). She was started on DKA management as per DKA protocol and also started on broad spectrum of antibiotics with vancomycin and meropenem for pneumonia.

**Figure 3:**
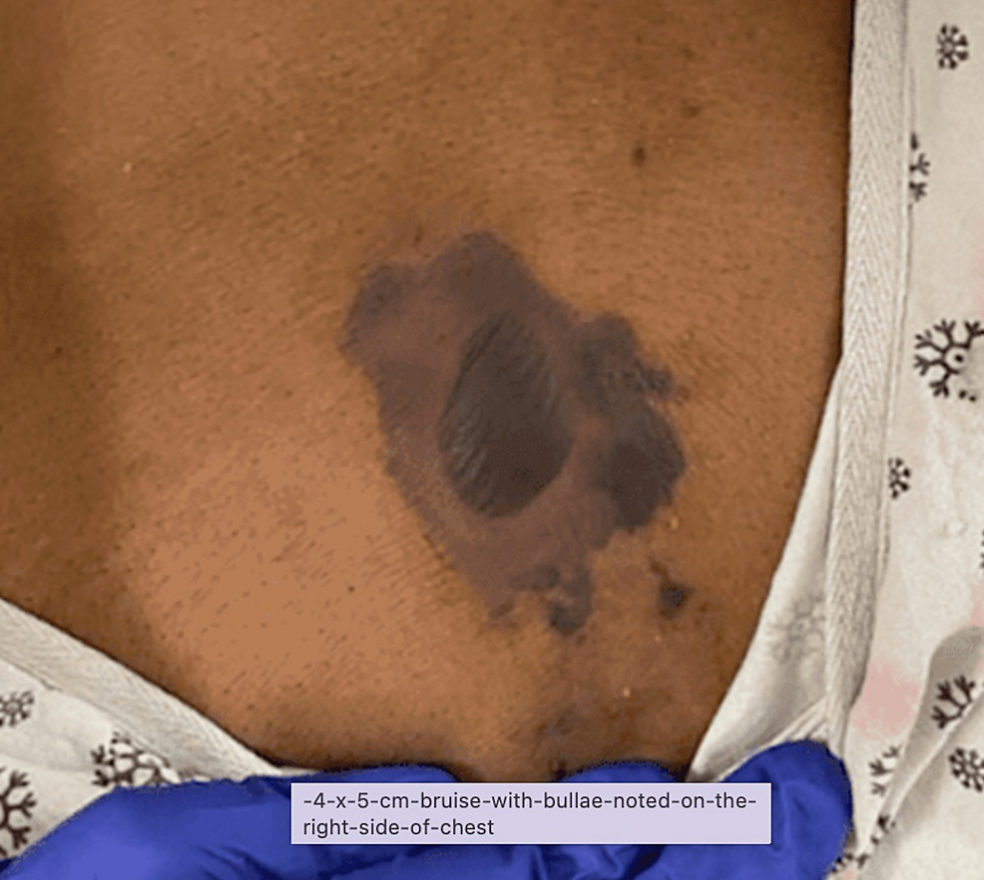
4 x 5 cm bruise with bullae noted on the right side of chest

**Figure 4:**
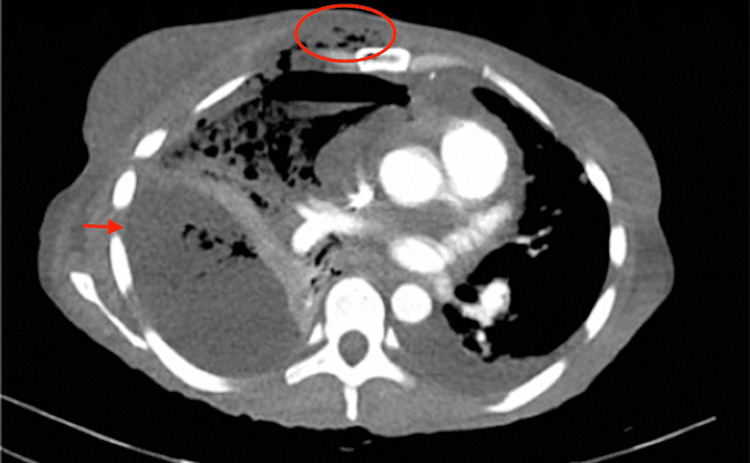
Large right pleural effusion with multiple air-fluid levels (red arrow) and subcutaneous emphysema (red circle) involving the anterior right chest wall.

On day 2 of admission, the patient was found in distress with tachycardia and a 4 x 5 cm bruise with bulla noted on the right side of the chest (Figure 3). The surgery team recommended continuing intravenous antibiotics. CT of the chest revealed large right pleural effusion with compressive atelectasis of the right lung. Right pleural space demonstrates multiple air-fluid levels. Findings are associated with subcutaneous emphysema involving the anterior right chest wall. Alveolar opacity at the left upper lobe and left lower lobe compatible with consolidations. Small left pleural effusion (Figure 4) and CT of the abdomen revealed pancreatic calcifications as well as a pancreatic pseudocyst on the tail measuring 5 x 7.2 cm.

A 32 French chest tube was placed immediately for both diagnostic and therapeutic tap and 600 ml of cloudy pleural fluid was removed and sent for analysis. On the same day due to worsening respiratory status, the patient was intubated electively. Pleural fluid analysis showed exudative type of effusion with criteria met for the empyema: WBCs 10,000 cells/ul with neutrophil 66% and lymphocyte 35%, red blood cells (RBCs) 5950 cells/ul, pH of 6.9, lactate dehydrogenase (LDH) 5865I U/L, protein 4.4 g/dl, glucose <2 mg/dl. Pleural fluid Gram stain and culture were negative. Three samples of acid-fast bacteria (AFB) stain and Mycoplasma were negative.

**Figure 5:**
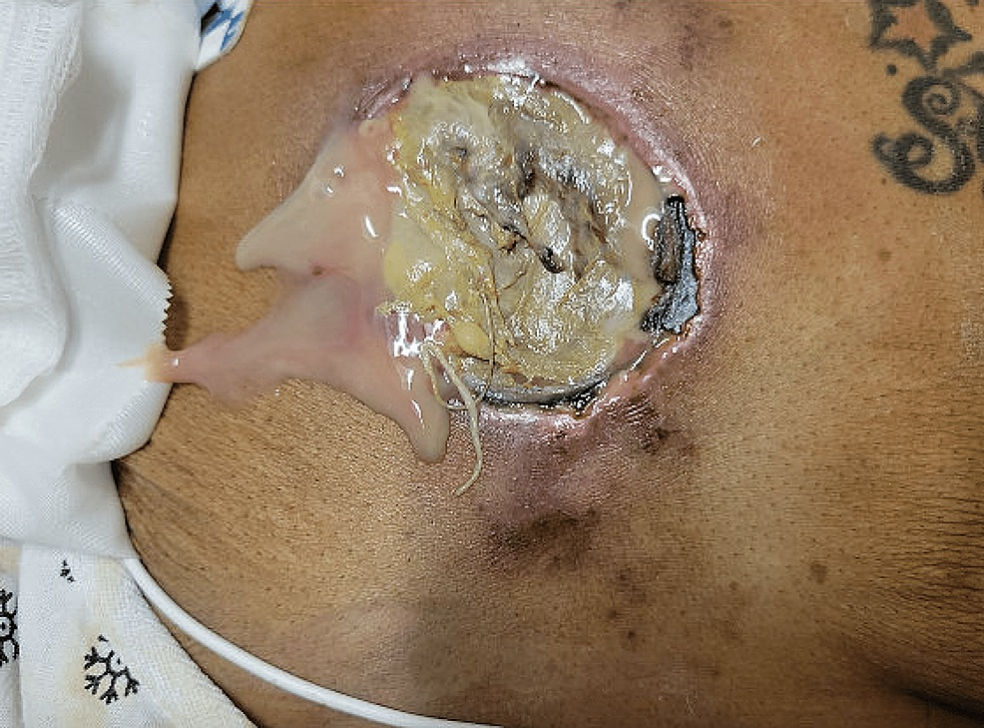
Skin ulcer measuring 4X6 cm with irregular borders with purulent drainage

**Figure 6:**
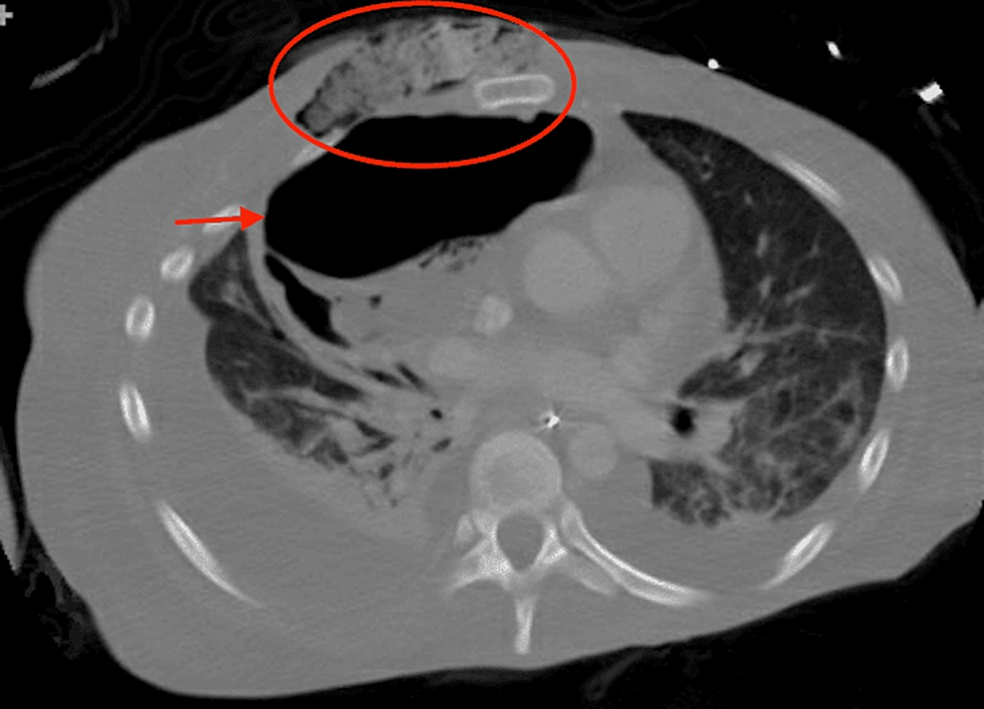
Enlarging anterior 14 x 10 cm pleural air collection (red arrow) probably related to bronchopleural fistula resulting in compressive atelectasis of the right upper and middle lobes, as well as resulting in left mediastinal shift. Right paramedian 11 x 8 cm full-thickness anterior wall soft tissue ulceration (red circle) with fistulous communication with the right pleural air collection.

During the hospital course the chest wound further progressed to an ulcer measuring 4x6 cm with irregular borders (Figure 5). The borders exposed subcutaneous tissue and partly necrotic borders and air bubbling with breathing likely due to development of bronchocutaneous fistula. Immediate debridement and evacuation of the complex abscess were done by cardiothoracic surgery. Repeat CT chest revealed enlarging anterior 14x10 cm pleural air collection related to bronchopleural fistula as well as right paramedian 11x8 cm full-thickness anterior wall soft tissue ulceration with fistulous communication with right pleural air (Figure 6). 

The following week, she was taken to the operating room (OR) by cardiothoracic surgery and found to have extensive purulent and fibrotic empyema involving the entire pleural cavity. Decortication of the right lower lobe and right middle lobe and debridement of the anterior chest wall with irrigation of the pleural cavity was performed. The right upper lobe had already started developing fibrosis so drainage of abscess was only done. Another chest tube with wall suction was placed. The patient was started on Ancef and Flagyl. The patient's wound biopsy result came positive for Mucor. Amphotericin B and isavuconazole started immediately. A tracheostomy was also performed followed by weaning off of the ventilator.

A week later patient was again taken to OR for debridement of chest cavity, resection of right ribs 2-6, repair of multiple bronchopleural fistula, right chest wall reconstruction with latissimus myocutaneous flap, and application of Acell graft had been completed. Later her hospital course was complicated by acute thrombosis of right internal jugular (IJ) and subclavian axillary, followed by cardiac arrest and eventually patient expired. 

## Discussion

Mucormycosis is an angioinvasive fungal infection within the subphylum Mucoromycotina belonging to the order Mucorales [1,6]. It’s abundant in soil that is associated with decaying leaves, rotten wood, animal waste, and even mucus [1,7]. Rhizopus is the most prevalent genus responsible for mucormycosis, followed by Mucor and Lichtheimia [1]. The route of infection in humans is inhalation which transmits spores to the lung or paranasal sinuses [7,8] and the inoculation of fungus into skin abrasions or wounds [1]. Immunocompetent patients can be affected as well if fungus spores are directly inoculated in the skin as a result of trauma or burns. This fungus can survive in glucose-rich mediums, especially in poorly controlled diabetic patients [8]. Diabetes/DKA temporarily disrupts the ability of transferrin to bind iron, and this alteration eliminates a significant host defense mechanism and permits the growth of Mucor/Rhizopus [9]. Mucormycosis was initially documented in 1876 by Fürbinger in Germany, who described a patient who died of cancer and had a hemorrhagic infarct with fungal hyphae and a few sporangia in the right lung [6]. Geographically, risk factors for mucormycosis differ substantially. DM is the most prevalent underlying disease worldwide, particularly in low- and middle-income nations like India, Iran, Mexico, and nations in the Middle East and North Africa. Hematological malignancies and transplantation are the most prevalent underlying illnesses in developed countries like Europe, USA and Australia. Other risk factors for mucormycosis include corticosteroids, other immunosuppressive agents and iron overload as iron plays a crucial role in the pathogenesis of this infection. Other disorders related to mucormycosis include intravenous drug abuse, AIDS, renal failure, liver ailments, persistent alcoholism, malnutrition, and low birth weight newborns.

The incidence of mucormycosis is increasing, however the actual incidence/prevalence is unknown due to a lack of population-based investigations. Mucormycosis primarily affects immunocompromised people whose immune systems are unable to combat the fungi [1]. Infection of medical devices, ventilation systems, and hospital disposables such as bandages and hospital linen has also been related to mucormycosis epidemics [1]. It is mostly diagnosed through laboratory examination of a biopsy taken from the infected location; other imaging procedures such as CT are also useful for diagnosis [1]. It is classified as rhinocerebral, pulmonary, cutaneous, gastrointestinal, disseminated, or other, depending on the clinical presentation, which includes unusual rare types such as endocarditis, osteomyelitis, peritonitis, renal, etc.

During the COVID-19 pandemic, there was an inflow of patients with ketoacidosis and hyperglycemia due to corticosteroid treatment, leaving patients more prone to fungal infection [8,10]. COVID-19 may increase mucormycosis risk due to COVID-19-induced immunological dysregulation or associated treatments such as corticosteroids and immunomodulatory medications (e.g. tocilizumab or baricitinib) that decrease host defenses against molds [11]. This is an uncommon yet life-threatening fungal illness and was responsible for 41,512 cases and 3,554 deaths between May 5 and July 12, 2021. The majority of these cases occurred during ongoing severe acute respiratory syndrome coronavirus 2 (SARS-CoV-2) outbreaks in India, forcing the Central Government of India to proclaim a mucormycosis epidemic on May 10, 2021 [10].

Without early aggressive interventions such as antifungal therapy (amphotericin B) and debridement of dead tissue, the disease often progresses rapidly and results in death [1,10]. Early detection has been demonstrated in studies to improve survival and lessen the need for the extent of surgical resection, disfigurement, and pain. Prior to COVID-19 the overall mortality rate of mucormycosis was 54%, and individuals with disseminated mucormycosis had the highest mortality rate of 96%, followed by those with lung (76%) and sinus (46%) infections [1,10]. The type of mold, infection site(s), underlying condition of the patient, or recent history of disease, such as COVID-19, all influence the infection's outcome [10].

Delaying treatment and diagnosis can result in unmanageable complications such as cavernous sinus thrombosis, disseminated infection, osteomyelitis, and ultimately death [3]. A multifaceted approach should invoke both clinical expertise in antifungals, surgical consults, and treatment of underlying conditions [1]. Mucormycosis often leads to a poor prognosis because of misidentification at an earlier stage [3]. It is imperative to conduct routine microscopy to identify mucor in aspirates, biopsies, and cultures especially in lower and middle income countries [3]. Improved hygienic and sanitary controls would further prevent the spread of this fungal infection [4]. Reduced risk factors, surgical removal of injured tissue, and antifungal medicines like amphotericin B are all part of standard therapy [3]. Clinicians utilize a risk-based strategy for patients at risk of mucormycosis that considers immunosuppressive and steroid use, diabetes mellitus rampancy, COVID-19 extensiveness, and disease epidemiology [4].

## Conclusions

Mucormycosis is a rare invasive fungal illness that has become more common as the pandemic has progressed. COVID-19 medication and the virus's immunosuppression can leave patients vulnerable to Mucor infection. Patients with diabetes and immunosuppression, such as our patient, are at an even higher risk. We should be on the lookout for this infection because early detection is linked to a better prognosis. When COVID-19 patients are diagnosed, timely treatment and surgical treatments, careful monitoring of glycemic levels, and judicious use of corticosteroids, as well as proper hygiene and sanitation measures, all help to minimize the development of this fungal infection.
